# An ELISA-based method for detection of rabies virus nucleoprotein-specific antibodies in human antemortem samples

**DOI:** 10.1371/journal.pone.0207009

**Published:** 2018-11-07

**Authors:** Susan Realegeno, Michael Niezgoda, Pamela A. Yager, Amrita Kumar, Laboni Hoque, Lillian Orciari, Suryaprakash Sambhara, Victoria A. Olson, Panayampalli Subbian Satheshkumar

**Affiliations:** 1 Poxvirus and Rabies Branch, Centers for Disease Control and Prevention, Atlanta, Georgia, United States of America; 2 Immunology and Pathogenesis Branch, Influenza Division, Centers for Disease Control and Prevention, Atlanta, Georgia, United States of America; Wistar Institute, UNITED STATES

## Abstract

Rabies is a fatal encephalitic disease in humans and animals caused by lyssaviruses, most commonly rabies virus (RABV). Human antemortem diagnosis of rabies is a complex process involving multiple sample types and tests for the detection of antibodies, antigen (protein), and nucleic acids (genomic RNA). Serological diagnosis of human rabies includes the detection of either neutralizing or binding antibodies in the cerebrospinal fluid (CSF) or serum samples from unimmunized individuals without prior rabies vaccination or passive immunization with purified immunoglobulins. While neutralizing antibodies are targeted against the surface-expressed glycoprotein (G protein), binding antibodies to viral antigens are predominantly against the nucleoprotein (N protein), although there can be antibodies against all RABV-expressed proteins. To determine N protein-specific antibody responses in the CSF and serum during RABV infection, we developed an enzyme-linked immunosorbent assay (ELISA) with purified recombinant N protein expressed in *E*. *coli*. N protein-specific immunoglobulin (Ig) subtypes IgG and IgM were detected in the CSF or serum of previously diagnosed human rabies cases. In addition, anti-N protein seroconversion was demonstrated over the course of illness in individual rabies cases. We compared the N protein ELISA results to those of an indirect fluorescent antibody (IFA) test, the current binding antibody assay used in diagnosis, and show that our ELISA is consistent with the IFA test. Sensitivity and specificity of the N protein ELISA ranged from 78.38–100% and 75.76–96.77% with respect to the IFA results. Our data provide evidence for the use of an N protein ELISA as an additional option for the detection of RABV-specific IgG or IgM antibodies in human CSF or serum specimens.

## Introduction

Rabies is a viral disease that results in acute encephalitis in both human and animals. It is caused by neurotropic lyssaviruses, such as RABV, a single-stranded negative sense RNA virus belonging to the *Lyssavirus* genus and *Rhabdoviridae* family [1]. The genome of RABV encodes five proteins, including the N protein, which along with phosphoprotein (P) and RNA-dependent RNA polymerase (L) encapsulates the genomic RNA to form the ribonucleoprotein complex (RNC). The core RNC is surrounded by the viral envelope consisting of G and matrix (M) proteins. Immunogenicity to RABV has been reported primarily against G, N, or the RNC, which is mostly comprised of N protein [2–5]. Previous studies have demonstrated that immunization with either G or N proteins expressing recombinant vectors can offer protection against RABV infection in animal models [6–8]. In addition, N protein can enhance the adaptive immune responses, including neutralizing antibody activity [3, 9, 10]. Thus, G and N proteins are both ideal target antigens for measuring antibody responses following RABV vaccination or infection.

Detection of antibodies against RABV is an important indicator of immune and exposure status. Presence of anti-RABV antibodies in the CSF or in the serum of an unvaccinated individual is considered a positive human antemortem diagnosis for rabies [11], provided no passive immunization with intravenous immune globulin containing anti-RABV antibodies has occurred [12]. The two methods currently used for determination of anti-RABV antibodies are the rapid fluorescent focus inhibition test (RFFIT) [13] and the IFA test [14]. The RFFIT is a functional assay in which the ability of antibodies to inhibit RABV infection is determined. Since G protein is responsible for RABV entry and fusion, RFFIT detects only G protein-specific antibodies [15, 16]. The IFA test, on the other hand, provides information on binding antibodies (including non-neutralizing) which can be targeted against any of the RABV encoded proteins, although it is demonstrated to be predominantly against the G and N proteins [17]. The IFA has the advantage of being able to differentiate Ig subclasses, such as IgG or IgM, the latter being an indicator for recent or primary infection. Both of these methods require the use of infectious virus and a trained technician to visualize and recognize RABV-specific staining patterns. Alternatively, several ELISA based investigative antibody detection methods have been developed. While most ELISA methods use the G protein as the preferred target antigen [18–22], few have used the N protein [23–26]. However, the utility of these ELISA tests in human rabies antemortem diagnosis has not been demonstrated.

The RABV N protein is the most transcriptionally abundant protein during infection [27–29] and is typically identified in infected tissues in the form of inclusions [30]. Because of its high expression level, antigen detection diagnostic tests, like the direct fluorescent antibody (DFA) test and direct rapid immunohistochemical test (DRIT), utilize polyclonal or monoclonal antibodies (mAbs) against RABV N protein [31–33]. In addition, antigenic typing also involves the use of epitope-specific mAbs against the N protein to identify RABV-specific variants from infected specimens [34, 35]. Similarly, the intracytoplasmic inclusion staining patterns seen in the IFA test, which utilizes RABV-infected cells for detection of RABV-specific binding antibodies, resembles that of N protein [29, 36]. Therefore, N protein is an ideal target antigen for use in a binding antibody detection assay.

Here, we developed an N protein ELISA and investigated the immune response to RABV in specimens collected from previously diagnosed human rabies cases. These results were compared with the IFA test, the current binding assay used in the antemortem diagnosis of rabies. Similar to the IFA test, the N protein ELISA was able to detect both IgG and IgM subclasses in CSF and serum samples from individuals positively diagnosed with RABV infection. We propose that the N protein ELISA can be an additional tool for antibody detection of RABV-specific antibodies in human CSF and serum samples. The advantages of the ELISA are the ability to use recombinant protein and a colorimetric detection method without the need for infected samples or a fluorescence microscope, features that can be suitable for resource limited environments.

## Materials and methods

### RABV N protein expression and purification

Purified N protein was obtained from ARVYS Proteins, Inc involving the following protocol. In brief, the N gene sequence for RABV CVS (Challenge virus standard) 11 strain was codon optimized and chemically synthesized. It was cloned into pET100D expression vector (Thermo Fisher Scientific) with an N-terminal Histidine tag for efficient purification. The plasmid was transformed into *E*. *coli* DE3 Star expression cells, culture was subjected to cell lysis and the soluble fraction was purified using Ni-affinity chromatography. Purity of the N protein sample was evaluated in a 4–12% Bis-Tris polyacrylamide gel (Thermo Fisher Scientific) followed by staining with Imperial protein stain (Thermo Fisher Scientific) according to manufacturer’s instructions.

### Ethics statement

Historic patient specimens received at the CDC Rabies Laboratory for antemortem rabies diagnostic testing were used in the validation of the N protein ELISA. The samples originated from facilities in the United States (U.S.) where the patients were being hospitalized and treated. Samples were de-identified according to IRB approved CDC protocol #7028. A formal request to waive consent was approved by the CDC IRB.

### Clinical specimens

CSF and serum specimens collected from patients (January, 2005 to January, 2018) suspected of rabies were originally tested by the IFA test and RFFIT for binding and neutralization antibody detection, respectively. A total of 65 serum specimens and 46 CSF specimens from 16 rabies-diagnosed cases were tested in our study along with 50 sera and 30 CSF from rabies negative human rule-out patients, including patients exhibiting acute progressive encephalitic symptoms of unknown etiology. However, in some instances sample volume was limited or previous sample information was missing and only a subset of samples were included in a particular analysis. Samples from rabies and non-rabies cases were heat inactivated at 56°C for 45 minutes and evaluated for anti-N antibody detection by ELISA. Healthy sera from four unvaccinated individuals and one serum sample from a non-rabies case with a confirmed alternative diagnosis were used as controls. These samples were confirmed negative by both the IFA test and RFFIT prior to use. CSF negative controls were from non-rabies cases, in which there was an alternative diagnosis and samples were confirmed negative by the IFA test prior to use. Positive samples that were used as internal assay controls included standard rabies immune globulin (SRIG, Lot R-3) diluted to 2 IU/ml and an IFA positive human serum sample from a previous rabies positive case.

### Indirect fluorescent antibody (IFA) test

The IFA test was performed on clinical samples as part of rabies antemortem diagnostic testing at the CDC according to Clinical Laboratory Improvement Amendments of 1988 (CLIA) standards. For confirmation, repeat testing was done on a selected number of samples using the IFA protocol as described previously [37]. In brief, mouse neuroblastoma (MNA) cells (0.5x10^6^ cells/ml) were infected with RABV CVS-11 to obtain a target of 25 to 50% infected cells. The virus-cell suspension was plated drop-wise onto 4-well 6 mm Teflon^®^-coated microscope slides (Thermo Fisher Scientific) using a disposable dropper, yielding 1 drop per well. Slides were incubated in a moist chamber at 37°C with 0.5% CO_2_ for 20 hours to allow for infection and cell adherence. After incubation, wells were rinsed with 1x 0.01 M PBS and fixed with cold acetone for 30 minutes. Infection was confirmed by immunofluorescence using a monoclonal anti-N protein fluorescein isothiocyanate (FITC)—conjugated antibody (Fuijirebio Diagnostics, Inc) and visualized using an Axio Imager A1 fluorescence microscope (Zeiss) at 200X magnification. Heat-inactivated diluted serum or undiluted and diluted CSF were added to the wells and incubated for 30 minutes at 37°C, then rinsed twice with 1x PBS for 5 minutes. The secondary FITC-conjugated antibodies, goat anti-human IgG (H+L) (SeraCare) or anti-human IgM (SeraCare), were added and incubated at 37°C for 30 minutes, then rinsed twice for 5 minutes with 1x PBS. The slides were mounted with 20% glycerol and visualized with a fluorescence microscope. If no specific RABV inclusions were detected in the sample and the negative control, the sample was reported to have a negative IFA result. If the sample had FITC fluorescent staining of typical inclusions within cells as demonstrated in the positive control, the test was reported as positive.

### Confocal microscopy and imaging

Slides were prepared similarly to the IFA test procedure, in which a suspension of MNA infected cells were seeded on Teflon^®^-coated slides to make a monolayer, fixed with acetone and used for further staining. Fixed infected cells were blocked with 10% fetal bovine serum (FBS) in 1x PBS for 15 minutes at room temperature (RT). Mouse anti-N mAbs and human sera were diluted in 1x PBS with 10% FBS and incubated alone or in combination for 30 minutes at RT. Wells were then washed with 1x PBS multiple times. Alexa Flour 594 conjugated anti-mouse (Molecular Probes) and FITC-conjugated anti-human IgG (SeraCare) in 1x PBS were used as secondary antibodies. Secondary antibodies were incubated for 30 minutes at RT. Cell nuclei were stained with 4’, 6-diamidino-2-phenylindole (DAPI). The cells were then fixed with 4% paraformaldehyde for 10 minutes, followed by mounting with Prolong Antifade Mounting Media (Thermo Fisher Scientific). The cells were then visualized using a LSM 710 inverted confocal microscope (Zeiss).

### N protein IgG & IgM ELISA

Immunlon II high binding microtiter 96-well plates (Thermo Fisher Scientific) were coated with purified N protein diluted in carbonate-bicarbonate buffer (Sigma-Aldrich) overnight at 4°C. Protein coating concentration for IgG detection was 0.125 μg/ml and 0.50 μg/ml for IgM. The plates were washed three times with 0.05% PBS/Tween (PBS-T) between each incubation step and all incubations were at RT. Plates were blocked for 1 hour with blocking buffer (5% skim milk, 2% bovine serum albumin, and 2% goat serum in 0.05% PBS-T) at RT. CSF samples were diluted at 1:10 and serum samples were diluted at 1:100 in blocking buffer and incubated on the coated plate for 1 hour. Secondary goat anti-human IgG-horseradish peroxidase (HRP) conjugated antibody (SeraCare) was diluted in blocking buffer at 1:2000 and goat anti-human IgM-HRP conjugated antibody was diluted at 1:6000 (SeraCare), then added to the plates for 1 hour. To develop the samples, 3,3’,5,5’ Tetramethylbenzidine (TMB) 2-component substrate (Sera Care) was added to the plates, followed by TMB Stop Solution (SeraCare). The ODs were measured at 450 nm using the Enspire Plate Reader (PerkinElmer). In addition, two mouse anti-RABV N conformation-specific mAbs, clone CDC 62-15-2 and clone CDC 62-164-2 [38] were used as the primary antibody to detect specific epitopes using our ELISA format and anti-mouse IgG-HRP was used as the secondary detection antibody.

### Data analysis

Every plate contained two positive and three to five negative control samples. The negative control samples were used to determine the cutoff value (COV) for individual plates. All samples were tested in duplicate. The average OD value of the blank wells (blocking buffer only) was subtracted from control and test samples before the COV was calculated. The COV corresponded to the mean OD of all negative control samples plus three times the standard deviation. The COV was then subtracted from the average of each test sample’s OD value to determine positive and negative results, which were the reported OD values. Statistical analysis was performed on the calculated OD values from IFA positive and negative samples in GraphPad Prism software using the Mann-Whitney test. Statistics were reported as *P* values. Sensitivity and specificity percentages were calculated for each N protein ELISA test using the IFA results as the true positive and negative values. Sensitivity was calculated by the following formula (true positive/(true positive + false negative)). Specificity was calculated using the following formula (true negative/(false positive + true negative)). The 95% confidence intervals for sensitivity and specificity were also reported and are Clopper-Pearson confidence intervals.

## Results

### Rabies positive human serum detects N in RABV infected cells

**Fig 1:**
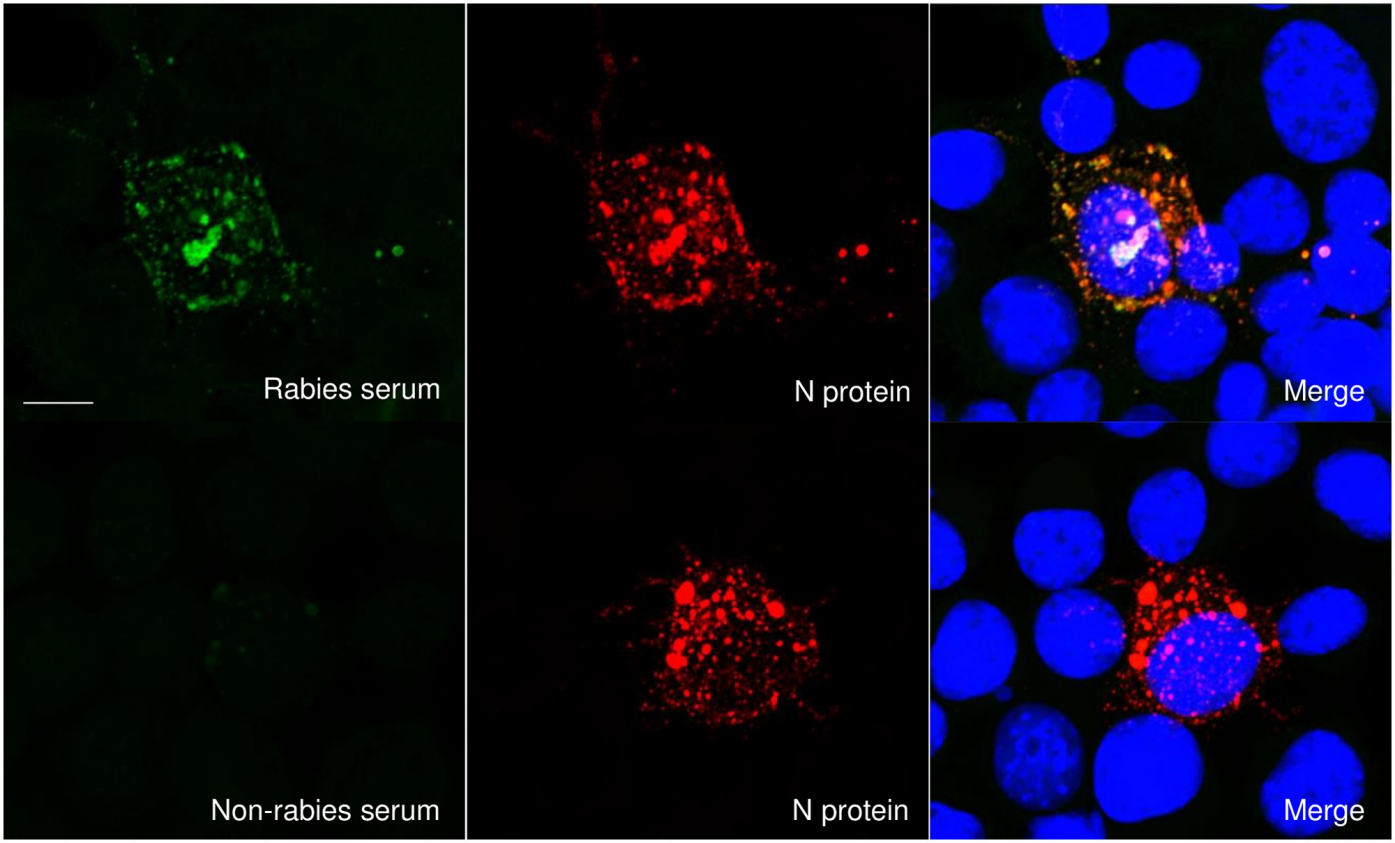
Antibodies in patient serum are able to detect RABV N protein. MNA cells grown on glass cover slips were infected with RABV CSV-11 strain for 20 hours. Antibodies present in a human serum samples from a rabies positive patient was used to detect rabies antigen (green, top left panel) and serum from a non-rabies case was used as a control (bottom left panel), followed by FITC conjugated anti-human IgG for visualization. RABV N protein (red) was visualized using anti-N protein mAbs (middle top and bottom), followed by Alexa 594 conjugated secondary antibody. Merged image of rabies serum with N protein is shown on the top right and merged non-rabies serum with N protein is shown on the bottom right. Merged images include the nucleus (blue), which was visualized using DAPI. Scale bar = 10μm. Original magnification: x40.

To demonstrate that the rabies positive human serum primarily detects N protein in an IFA assay, we first compared the IFA staining pattern of rabies positive human serum samples with that of anti-N protein staining in RABV-infected cells. RABV-infected cells were prepared on slides similarly to an IFA assay and incubated with either human rabies IFA positive serum or non-rabies IFA negative serum and mouse anti-N protein mAb. N protein and human serum staining were visualized by confocal imaging using anti-mouse IgG Alexa Flour 594 or anti-human IgG FITC conjugated antibodies. As expected, we observed specific staining with serum from a positive human rabies case and no staining with negative human serum (Fig 1, compare top and bottom left panels). The staining pattern observed with the positive serum localized with the anti-N protein mAb staining (Fig 1, merge), confirming the predominant detection of N protein by human serum in an IFA positive sample. This result suggested the potential use of an N protein-based ELISA for the detection of anti-RABV binding antibodies in human cases.

### Recombinant RABV N expression, purification and conformational analysis

**Fig 2:**
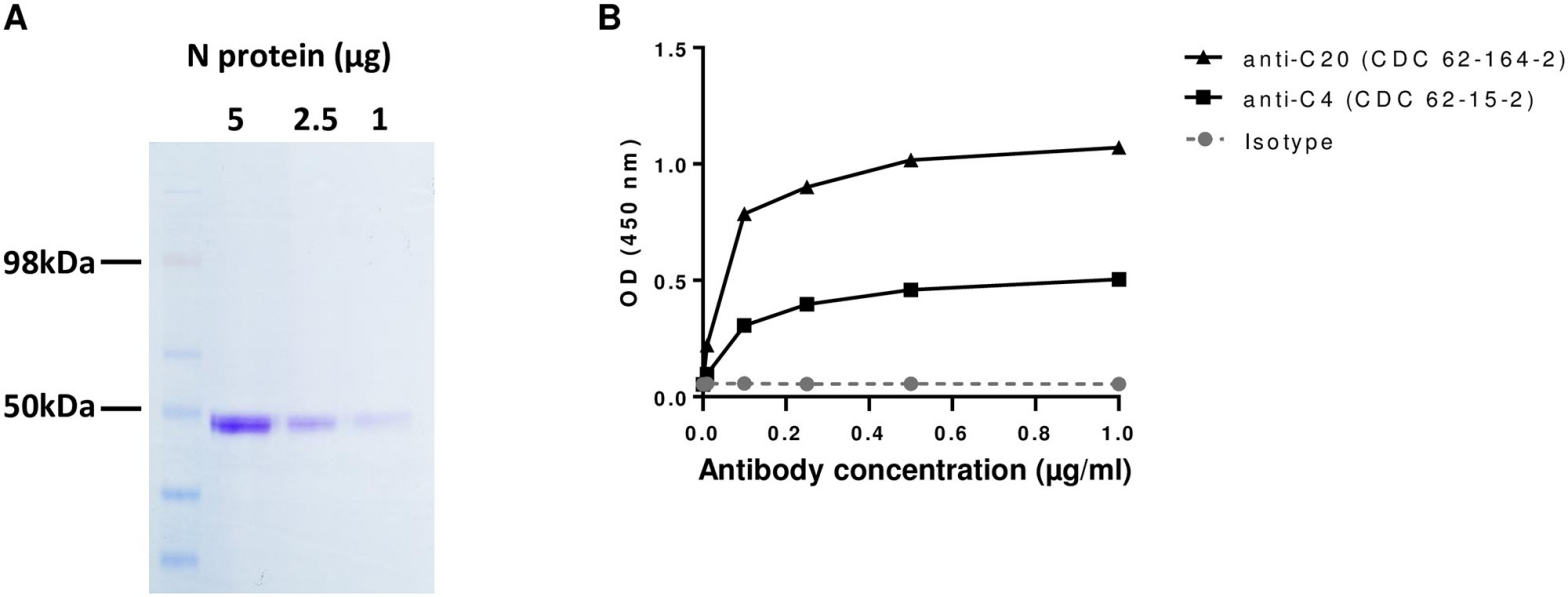
N protein expression purity and confirmation of native epitopes. (A) Recombinant N protein was separated on SDS-PAGE gel at indicated concentration and stained with Coomassie stain for visualization of all proteins in the N protein preparation. Protein ladder indicates estimated size of protein bands. (B) OD absorbance value of N protein detected with primary mAbs against N protein conformation epitopes at different antibody concentrations in an ELISA format. Mouse IgG isotype was used a control. Secondary antibody against mouse IgG conjugated with HRP was used.

Recombinant N protein from RABV CVS-11 was produced using an *E*. *coli* expression system. The overall purity of the N protein sample was evaluated by SDS-PAGE followed by staining with Imperial protein stain, a Coomassie blue staining for protein visualization (Fig 2A). The staining revealed a single prominent band at approximately 50kDa, suggesting that the N protein expressed was successfully isolated and was relatively free of other proteins.

To determine if the recombinant N protein retained native conformational epitopes, we used two mouse mAbs against N protein that were previously characterized to bind distinct conformation-specific epitopes [38–40]. Both mAbs demonstrated a concentration-dependent binding to recombinant N protein compared to the mouse IgG isotype control (Fig 2B). Since these mAbs are used routinely as primary antibodies for rabies antigenic typing in the IFA test, we are confident that the expressed N protein closely resembles native conformation. Hence, the recombinant N protein is a suitable antigen to detect binding of polyclonal antibodies generated in humans infected with RABV.

### N protein IgG and IgM ELISA optimization for serum and CSF

Using the purified recombinant N protein, we developed an indirect ELISA for the detection of IgG and IgM antibodies against RABV N protein in human CSF and serum specimens. The coating antigen concentration, sample dilution, and secondary antibody concentration were optimized using either SRIG, human sera, or CSF from rabies and non-rabies cases. SRIG, in addition to specimens from actual human cases, was used for optimization since it is a standardized preparation of rabies immune globulin with known concentration. Human specimens (serum or CSF) from rabies and non-rabies cases allowed for comparison of ELISA values to determine the background signal. We first determined the coating concentration and serum dilution for the IgG ELISA by narrowing our coating antigen range to 0.125–1.000 μg/ml and serum dilution from 1:100 to 1:400 for both positive and negative samples (S1A–S1C Fig). The ratio of positive (SRIG or rabies signal) to negative signal (non-rabies) was also calculated (S1D and S1E Fig). Since a decrease in positive to negative signal ratio was observed at increasing coating concentrations, 0.125 μg/ml of N protein and a serum dilution of 1:100 was used for subsequent experiments. Using a serum dilution of 1:100 optimized by the IgG ELISA, the N protein concentrations and anti-IgM secondary dilutions for the IgM ELISA were varied (S2 Fig). Since SRIG is not positive for the IFA IgM test, it served as a negative control in optimization, further substantiating the notion that detection of IgG antibodies are specific for N protein in the ELISA. The N protein coating concentration and secondary dilution that yielded optimal optical density (OD) measurements for the IgM ELISA was determined to be 0.5 μg/ml and 1:6000, respectively (S2 Fig).

To maintain consistency in our IgM and IgG ELISAs, concentrations as described above for coating and secondary antibody were used for CSF samples. The CSF sample dilution for both IgG and IgM was determined by titrating 3 rabies CSF positive samples and 2 non-rabies negative samples from 1:10 to 1:100 (S3 Fig). The background for CSF negative samples was relatively low at all dilutions. Therefore, to detect the largest difference in OD measurements between positive and negative samples and also capture any low positive samples, a 1:10 dilution of CSF samples were used for both IgG and IgM.

### Comparison of RABV N protein ELISA with the IFA test

**Fig 3:**
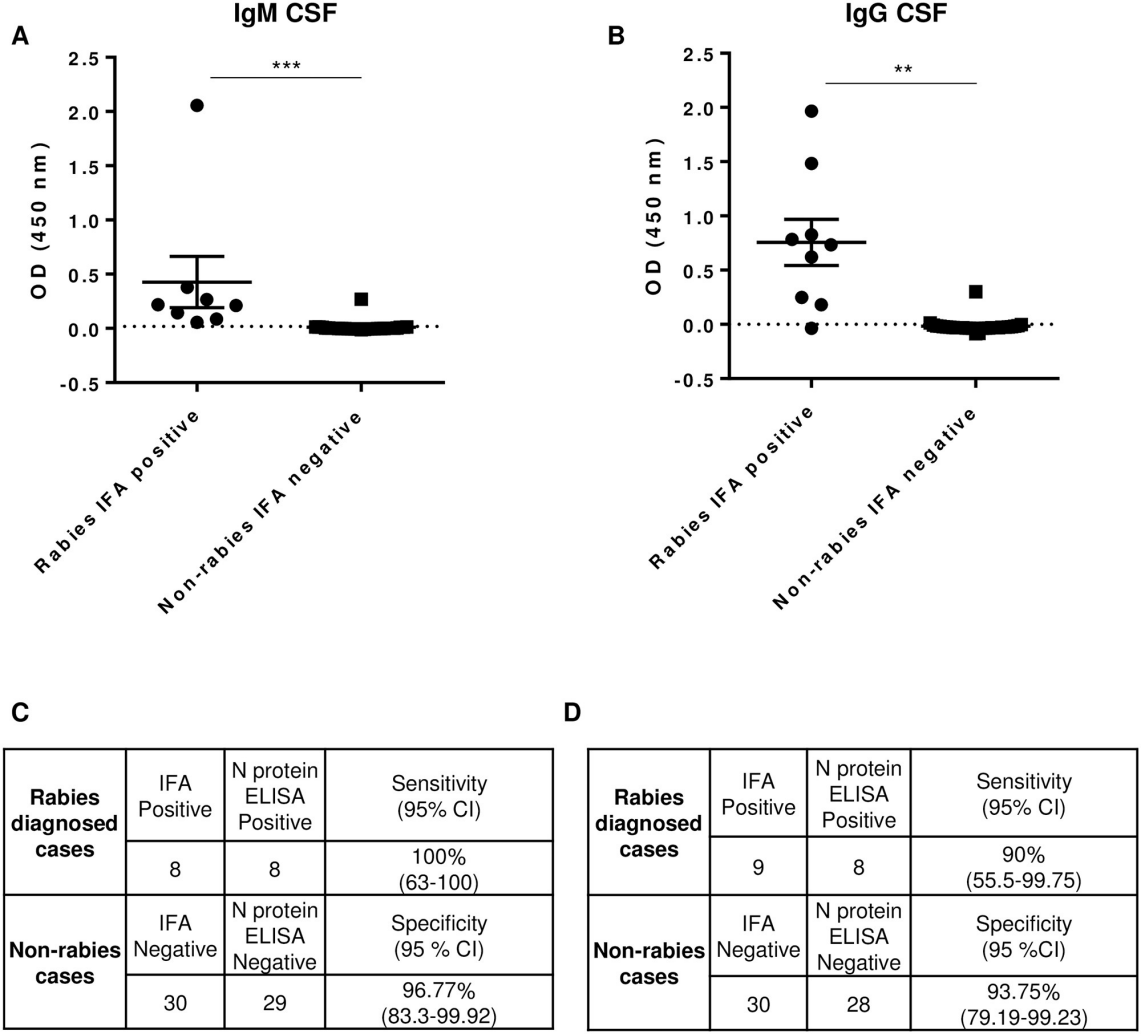
N protein IgG and IgM ELISA values in CSF relative to IFA result. ELISA values were determined by subtracting the COV from the OD values for each sample. (A) ELISA values for each IFA positive and negative CSF samples tested for IgM are shown. Values >0.02 are N positive. Statistics reported are with the exclusion of outlier data point. (B) CSF samples tested for IgG are shown. Values >0.00 are positive. IFA and ELISA results used to calculate sensitivity and specificity for (C) IgM and (D) IgG. The 95% confidence interval (CI) for each is shown. Mean and standard error are indicated by the error bars. *, P ≤ 0.01; **, P ≤ 0.001; ***, P ≤ 0.0001.

**Fig 4:**
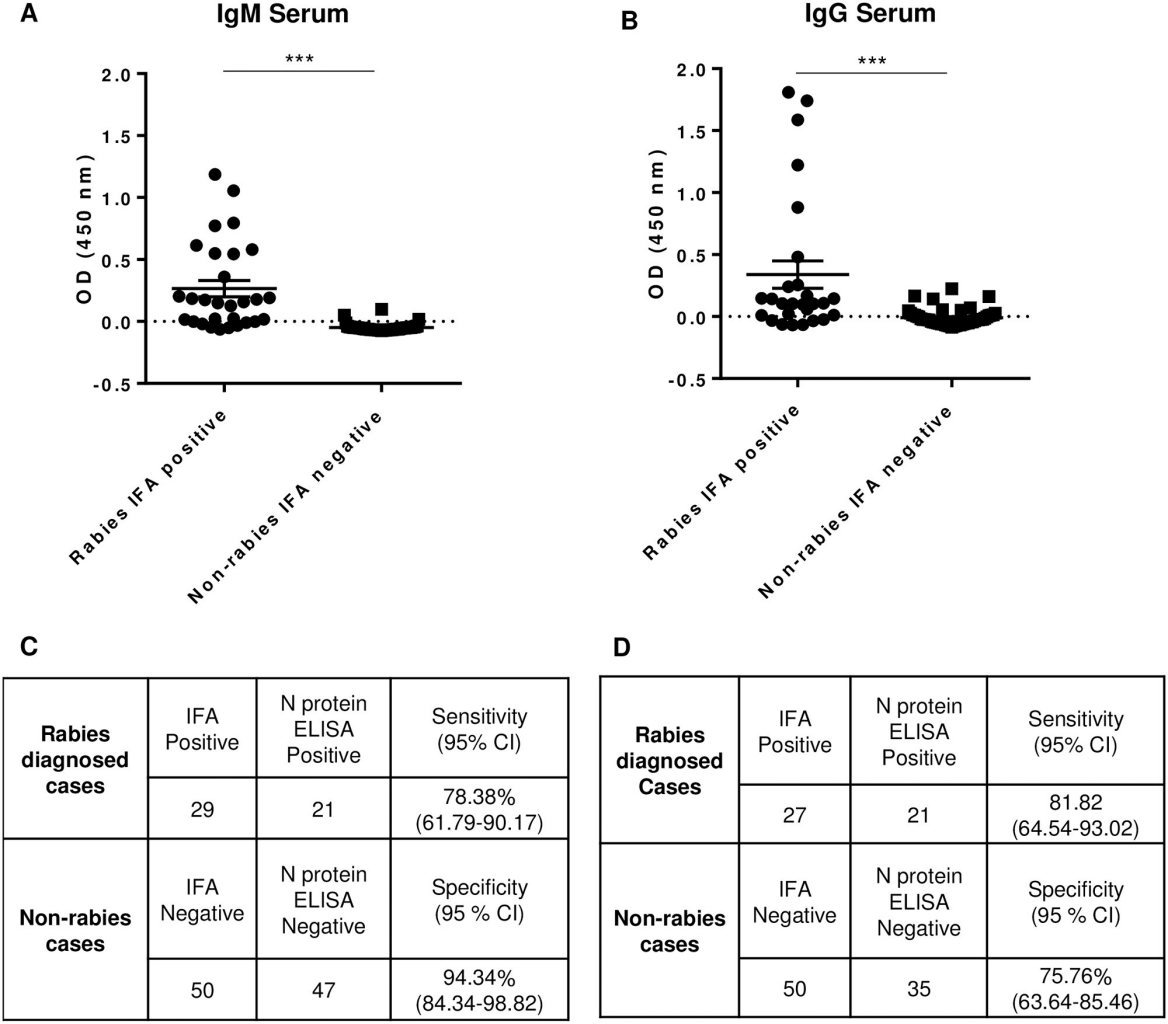
N protein IgG and IgM ELISA values in serum relative to IFA result. ELISA values were determined by subtracting the COV from the OD values for each sample. ELISA values for each samples for (A) IgM and (B) IgG are shown. Values >0.00 are ELISA positive. IFA and ELISA results are shown and used to calculate sensitivity and specificity for (C) IgM and (D) IgG. The 95% confidence interval (CI) for each is shown. Mean and standard error are indicated by the error bars. *, P ≤ 0.01; **, P ≤ 0.001; ***, P ≤ 0.0001.

Individual serum and CSF specimens from previous rabies and non-rabies diagnosed cases from 2005 to 2018 were tested for RABV N protein-specific antibodies using the N protein ELISA. A total of 77 sera and 39 CSF samples were tested for N protein IgG and 79 sera and 38 CSF samples were tested for N protein IgM, including the negative samples from non-rabies cases (S4 Fig). To check the stability of anti-RABV binding antibodies upon storage, IFA testing was repeated in a subset of samples and we observed consistent results to those previously reported. The cut off values (COV) were determined for each plate based on the OD values and standard deviation of the negative control samples. The OD values for both IgM and IgG rabies positive CSF samples varied widely in the ELISA (Fig 3A and 3B). Some samples had values just above the COV and others as high as 2.0, demonstrating differences in immune responses between the rabies cases. However, the average OD values for rabies CSF positive samples versus non-rabies negative samples was statistically significant for both IgM and IgG. Among the rabies IFA negative CSF samples, only three (1 IgM and 2 IgG) samples were higher than the COV value. The ELISA results for rabies positive serum samples also varied in terms of OD values (Fig 4A and 4B). Even though the average IgM and IgG OD values between positive and negative were statistically significant (Fig 4A and 4B), a number of false negative and false positive ELISA results were observed (Fig 4C and 4D).

The sensitivity and specificity of N Protein ELISA were calculated based on the IFA result as the true positive and negative. Although the readout of two assays is different (absorbance versus microscopy), there was a high correlation observed for both IgM and IgG in the CSF (Fig 3C and 3D). The sensitivity and specificity of CSF IgM ELISA for rabies and non-rabies cases were 100% and 96.77%, respectively when compared to the IFA result. Similarly, the CSF IgG ELISA had 90% sensitivity and 93.75% specificity. The sensitivity and specificity values for serum IgM and IgG results relative to the IFA results, however, were lower compared to the tests using CSF. The serum IgM ELISA exhibited 78.38% sensitivity and 94.34% specificity (Fig 4C), while serum IgG ELISA demonstrated 81.82% sensitivity and 75.76% specificity (Fig 4D). We presume one of the reasons for the higher proportion of discordant results in serum compared to CSF may be due to higher non-specific binding. Even in the IFA test, serum exhibits a higher background compared to corresponding CSF samples from a single patient.

### N protein ELISA results for rabies classification

**Fig 5:**
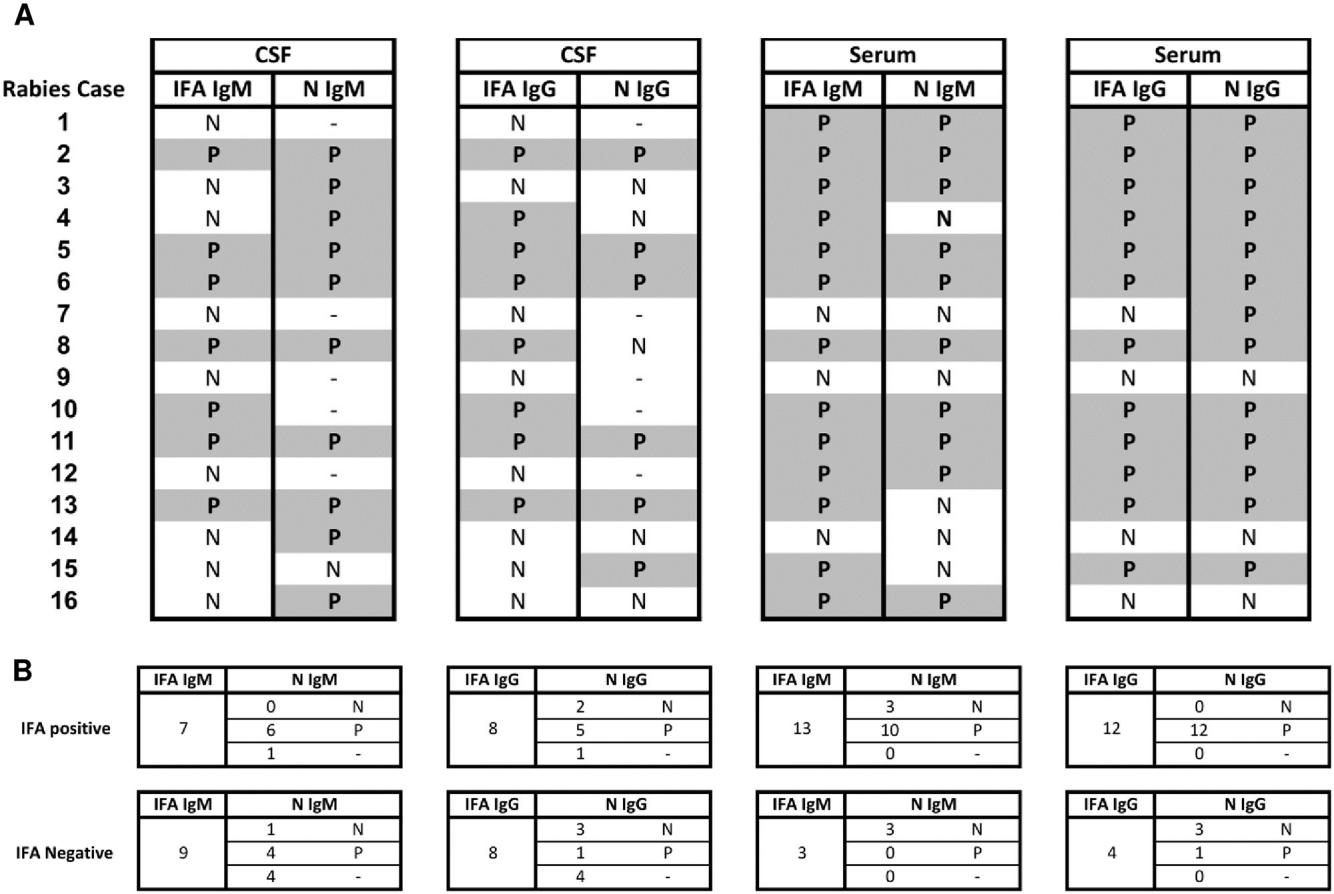
Detection of antibodies in CSF and serum of rabies positive cases. (A) A series of CSF and serum specimens from 16 diagnosed rabies cases (numbered 1–16) were tested for IgG and IgM N protein binding by ELISA and results were compared to IFA results. ‘P’ indicates the test result was positive, ‘N’ indicates the test was negative, and ‘−’ indicates no result could be obtained. (B) The total number of rabies cases with IFA positive or IFA negative results are listed for each IgM or IgG IFA test and the breakdown of how many of those were ELISA negative, positive, or had no data available due to sample availability are indicated.

We further evaluated the N protein ELISA by comparing the results to the IFA results of independent rabies cases. As previously mentioned, a rabies diagnosis is determined by testing a variety of samples with different methods. Hence, the outcome of the ELISA results does not change the overall interpretation of a previous rabies diagnosis. The IFA and ELISA results were compared in a total of 16 individual rabies cases with exposure to a rabid bat or dog via bite, contact, or unknown contact. The clinical presentation of each patient varied, with symptoms including encephalitis, fever, headache, agitation and combativeness, paresthesia, and hydrophobia (S1 Table). In this analysis, all samples from a single patient were treated as a set and we determined if the patient had at least one positive specimen for RABV antibodies by the IFA test or N protein-specific antibodies by ELISA during the course of the disease (Fig 5A). The number of specimens from a single rabies diagnosed individual ranged from 1 to 14 for either serum or CSF (S1 Table).

Seven rabies-diagnosed cases were reported to have an IgM response by IFA in CSF specimens and of those 7 cases, 6 cases had an N protein ELISA positive result, and 1 case we could not obtain data due to limited availability of sample volume (Fig 5B). Of the 9 cases that had a previous negative IgM IFA result in the CSF, N protein ELISA was positive for 4 cases, 1 case was negative and we could not obtain data for the remaining 4 cases. In terms of the IgG IFA results for CSF specimens, 8 cases were previously positive and 8 were previously negative. Of the 8 IFA positive cases, 5 were positive by IgG N ELISA, 2 were negative and for 1 sample we could not obtain data. For the IFA negative cases, N protein ELISA detected one positive, 3 negative, and 4 cases did not have sufficient sample to test. Overall, 10 of 11 cases (based on sample availability) were positive for IgM and 6 of 11 were positive for IgG using N protein-specific ELISA in the CSF.

For the serum, 13 rabies-diagnosed cases were positive for IgM by the IFA test and 3 cases were negative. For IgG, 12 cases were previously positive and 4 cases were previously negative for IgG by the IFA (Fig 5A). Of the 13 IFA IgM positive cases, 10 were positive by IgM N protein ELISA and 3 were negative (Fig 5B). From the 3 negative IFA IgM cases, all cases were also negative in the N protein ELISA. For serum IgG, of the 12 IFA positive cases, all 12 were also positive in the N protein ELISA and of the 4 IFA negative cases, 3 were negative and 1 was positive by N protein ELISA. Overall, 10 of 16 cases had N protein IgM seroconversion and 13 of 16 cases had N protein IgG seroconversion. The N protein ELISA results for serum from rabies-diagnosed cases was mostly consistent with previous IFA results and the N protein ELISA was able to detect an additional IgG positive serum sample (Fig 5).

### Kinetics of anti-N antibody detected in rabies-diagnosed cases

**Fig 6:**
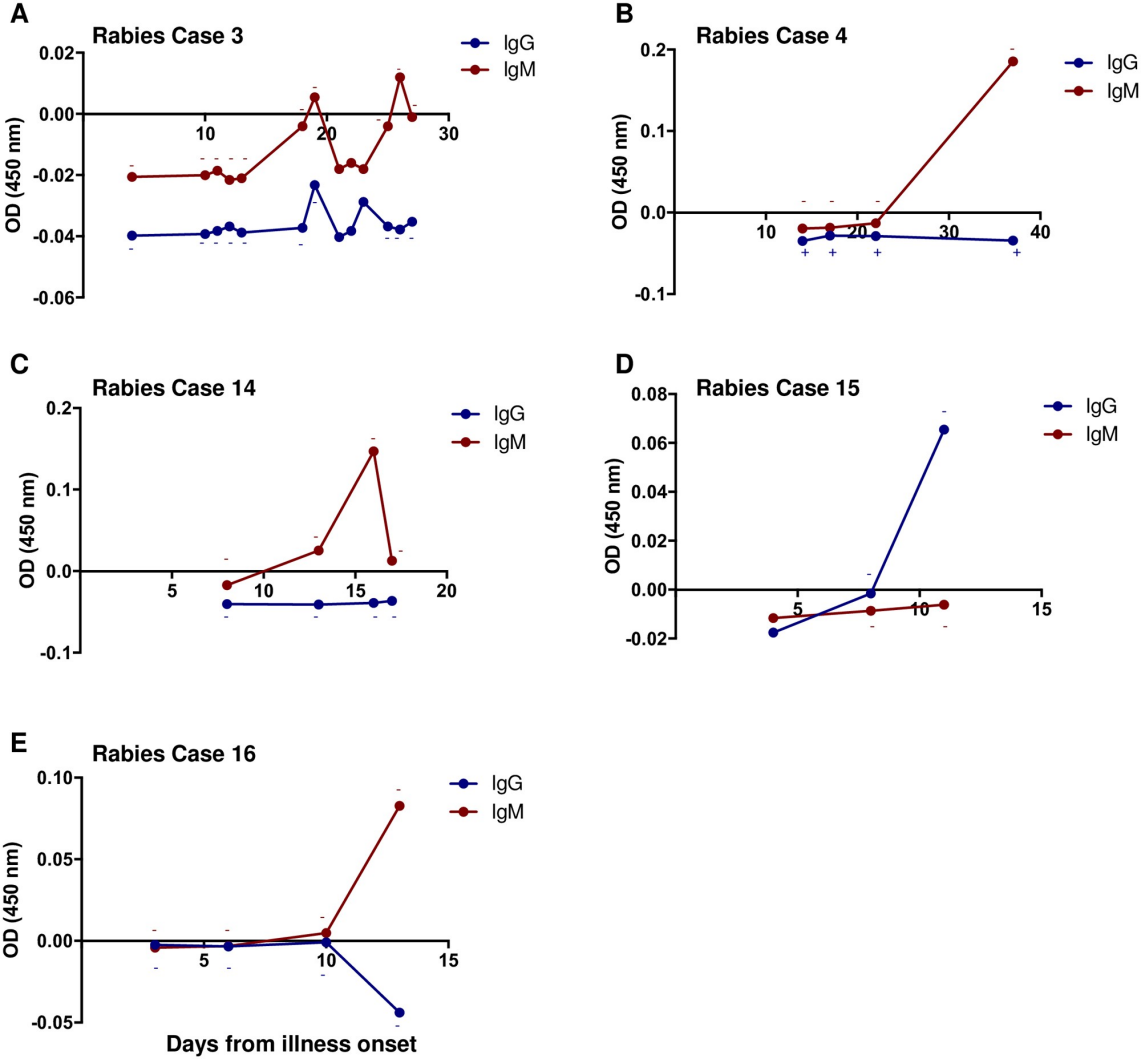
N protein antibodies over the course of illness in the CSF of rabies cases. N ELISA values were determined by subtracting the COV from the OD values for each sample. N protein IgG (blue circles) and IgM (red circles) ELISA values for a series of specimens from a single rabies case corresponding to Table 1A are shown. The corresponding IFA result for that particular sample is indicated by the (+) symbol for a positive result, (-) for a negative result, and no symbol indicates no IFA data was available for that sample. The color of the symbol corresponds to the IFA result for IgG (blue) or IgM (red). The x-axis indicates days from illness onset that the specimen was collected and the y-axis shows the ELISA value. (A) Rabies case 3 IgG and IgM N ELISA values. (B) Rabies case 4 IgG and IgM N ELISA values. (C) Rabies case 14 IgG and IgM N ELISA values. (D) Rabies case 15 IgG and IgM N ELISA values. (E) Rabies case 16 IgG and IgM N ELISA values.

**Fig 7:**
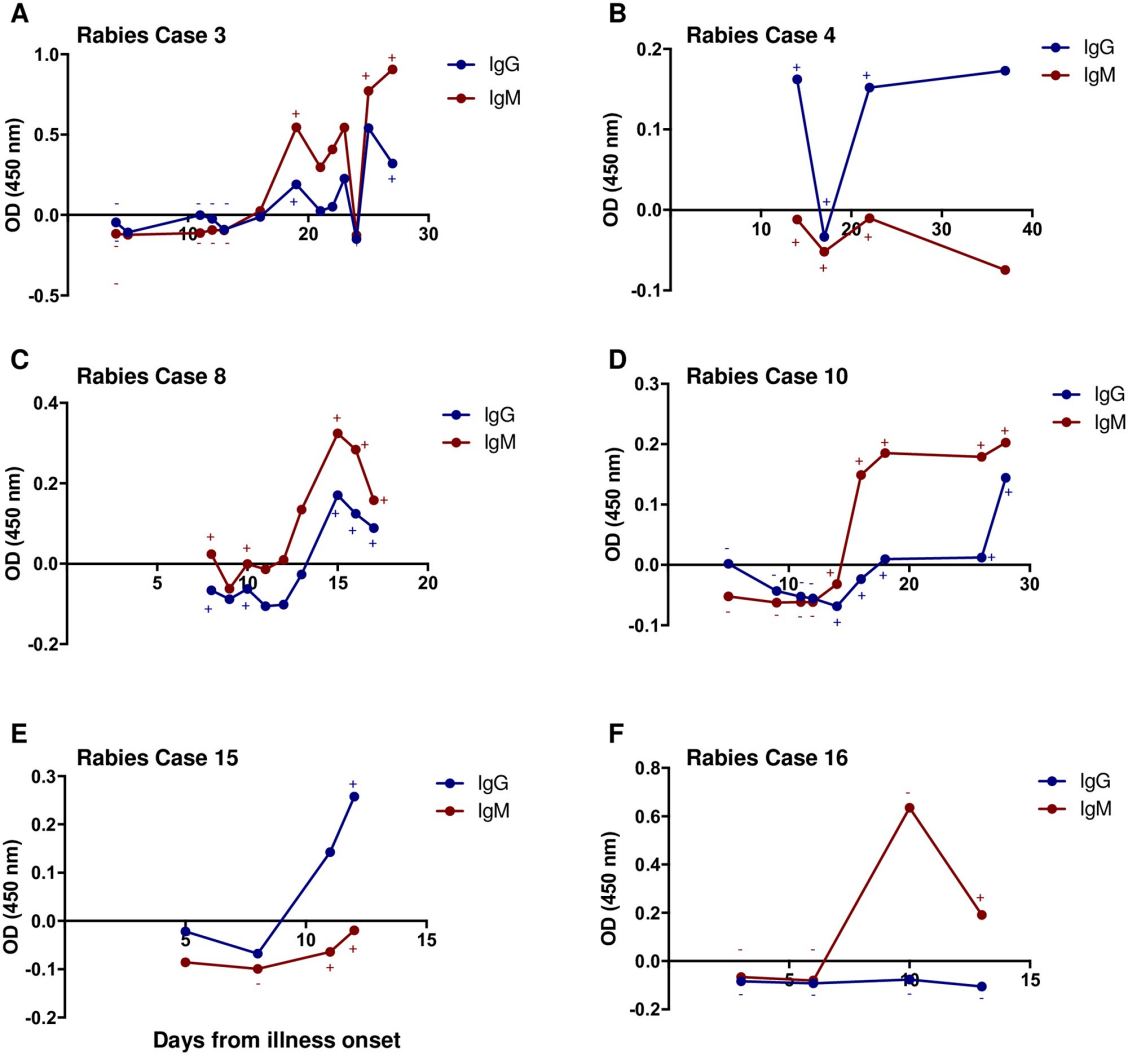
N protein antibodies over the course of illness in the serum of rabies cases. N ELISA values were determined by subtracting the COV from the OD values for each sample. N protein IgG (blue circle) and IgM (red circles) ELISA values for a series of specimens from a single rabies case corresponding to Table 1A are shown. The corresponding IFA result for that particular sample is indicated by the (+) symbol for a positive result, (-) for a negative result, and no symbol indicates no IFA data was available for that sample. The color of the symbol corresponds to the IFA result for IgG (blue) or IgM (red). The x-axis indicates days from illness onset that the specimen was collected and the y-axis shows the ELISA value. (A) Rabies case 3 IgG and IgM N ELISA values. (B) Rabies case 4 IgG and IgM N ELISA values. (C) Rabies case 8 IgG and IgM N ELISA values. (D) Rabies case 10 IgG and IgM N ELISA values. (E) Rabies case 15 IgG and IgM N ELISA values. (F) Rabies case 16 IgG and IgM N ELISA values.

We examined the kinetics of anti-N protein IgG and IgM antibodies in the CSF and serum of several individual rabies cases. A total of 5 cases for CSF and 6 cases for serum were evaluated based on days from illness onset to the sample collection date, which varied from case to case. Antibodies in the CSF were detected after day 10 of illness onset (Fig 6) and seen as late as Day 37. In all cases, only one of the two antibody subclasses was detected in CSF. For instance, in rabies cases 3, 4, 14 and 16, only IgM antibodies were detected (Fig 6A, 6B, 6C and 6E), whereas in case 15 only IgG antibodies were detected (Fig 6D). In contrast, the serum IgG and IgM profiles for 3 rabies cases showed seroconversion of IgM before IgG (Fig 7A, 7C and 7D) and 3 cases only IgM or IgG was detected (Fig 7B, 7E and 7F). The earliest serum antibodies were detected was on Day 8 (Fig 7C) and the latest was on Day 32 (Fig 7B). Interestingly, for rabies case 15, only IgG was detected in both CSF and serum, but no IgM. In cases 3 and 8, the onset and progression of IgM and IgG antibody profiles exhibited a similar pattern.

## Discussion

Antemortem diagnosis of rabies is challenging due to the clinical course of the disease that can be influenced by viral pathogenesis and immunological status [41]. Laboratory assays rely on the dissemination of the virus for the detection of nucleic acid, antigen, and RABV-specific antibodies, which can depend on intermittent viral excretion [42]. The World Health Organization (WHO) recommends the collection of several specimen types over the course of illness in order to perform a variety of standard diagnostic tests including those for virus isolation, antigen, RNA and antibody detection [42]. However, this becomes increasingly difficult in countries with limited resources to collect the appropriate samples and have the equipment to perform the recommended assays. Therefore, development and evaluation of accessible assays are important for determining the true burden of disease. Here, we evaluated an ELISA-based assay for detection of RABV N protein-specific antibodies in human serum and CSF specimens from previously diagnosed rabies cases.

Antemortem diagnosis of rabies at the CDC requires 4 specimen types (saliva, serum, CSF, and skin) [43] to perform antigen detection using DFA [31, 32], nucleic acid detection using RT-PCR [44–46], and antibody detection using IFA [37] and RFFIT [13]. RABV antibody detection is informative for determining immune status and previous exposure to RABV antigens. This information can be useful not only in the diagnosis of rabies but also in surveillance, vaccine immunogenicity or research studies. At CDC, the two methods used for antibody detection in human antemortem testing are the IFA test and the RFFIT. In addition, the fluorescent antibody virus neutralization (FAVN) test [43, 47, 48] and the ELISA are also standard human antemortem antibody detection tests suitable for serum and CSF [42, 47]. ELISA-based assays for determining antibody response after vaccination utilize predominantly G protein [18–22] as the target antigen since it is an exposed viral surface protein and highly immunogenic. However, viral N protein is also highly immunogenic and reported to enhance immunity and virus neutralizing antibody activity [3, 4, 10]. A few studies have investigated N protein-specific antibody responses in humans using N protein as the target antigen for ELISA-based assays [23–26]. Here, we developed an N protein ELISA and validated it using samples that were previously confirmed as positive or negative for the presence of anti-RABV binding antibodies by the standard IFA test. This study demonstrated RABV N protein-specific responses in human CSF and anti-N seroconversion with both IgG and IgM subtypes in a single patient over the course of illness using an N protein ELISA.

One of the advantages of performing a binding assay compared to a neutralization test is the ability to identify Ig subclass types, such as IgG and IgM. While IgG will generally indicate a previous exposure to RABV antigen, the IgM develops before IgG during the acute phase of a recent infection. Anti-RABV antibodies on average are detected in the serum or CSF of rabies patients on day 7 or 10.5, respectively, from illness onset and as early as 2 days [49]. The primary objective of this study was to investigate the use of recombinant N protein for binding antibody detection in clinical samples diagnosed with RABV infection. We developed an indirect ELISA to detect either IgM or IgG antibodies generated against RABV N protein following an infection in CSF and serum samples, which are two of the four sample types used in the antemortem diagnosis of rabies. We demonstrated detection of both IgM and IgG anti-N antibodies in CSF and serum in several previously diagnosed human cases and also show the kinetics of antibody detection in individual rabies cases in which multiple samples were available. From the cases that had detectable N protein-specific antibodies, there was variability in the time of illness onset to the time of antibody detection and also in the profiles of antibody response. For instance, in one case the IgM response in the CSF was detected as early as day 13 but reached a peak at day 16 and dropped by day 17. In another case, there was an IgG but no IgM response detected in the CSF, although the IFA test did not detect any CSF antibody responses. The kinetics of antibody responses in the serum was generally observed as expected. Detection of IgM was followed by IgG, but in two instances IgG was detected with no IgM. IgG detection in the absence of IgM has previously been reported for rabies [50]. However, it is possible that IgM was below the detection in both the ELISA and the IFA in these particular samples. In some instances, no antibodies were detected throughout the course of illness, especially in the CSF [49, 51, 52].

The kinetics of antibody production, in addition to antigen (N protein) and genomic RNA detection, varies in rabies cases. Therefore, performing a variety of tests can increase the likelihood of obtaining a positive antemortem diagnosis [49, 53]. Antigen detection (DFA) and genomic RNA (RT-PCR) -based assays are also important for rabies antemortem diagnostics and are performed as part of the standard diagnostic tests [42] in conjunction with antibody detection assays. Although antigen and genomic RNA can be detected initially in nuchal skin biopsy and saliva samples, antibodies in the serum or CSF may not be detected for several days after the onset of symptoms [41, 49, 50, 54]. In animal studies, generation of antibodies can enhance infection and results in ‘early death’ due to complement-mediated opsonization of immune complexes [55, 56], however, the role of immunopathology in positive human cases is not clearly defined. Conversely, in some cases of survival, patients had higher levels of neutralizing antibodies in the serum and CSF early on and antigen or genomic RNA was not detected in nuchal skin biopsy and or in saliva samples [50, 57–59]. This further emphasizes the importance of collecting multiple antemortem samples (nuchal skin biopsy, serum, CSF and saliva) for making a proper diagnosis and potential prognosis.

In this study, we compared the performance of N protein ELISA with IFA for detection of binding antibodies. In the N protein ELISA, CSF samples exhibited high sensitivity and specificity in both IgM and IgG assays (greater than 90%) when considering the IFA results as the true positive and true negative. The IgG and IgM ELISA tests using serum samples exhibited a lower sensitivity and specificity compared to the tests using CSF, but the N protein ELISA was still able to detect both IgM and IgG subclasses in a majority of rabies samples that were previously IFA positive. The discordance between the ELISA and IFA assays may be due to differences in assay conditions and the method of detection. While the IFA test can detect RABV-specific antibodies against all viral proteins encoded by RABV, the ELISA detects only N protein-specific antibodies. Therefore, a positive IFA and a negative ELISA result for a particular sample may suggest the presence of antibodies against other proteins, particularly G protein. In addition, the IFA test has the advantage of being able to visually examine the pattern for anti-RABV- specific versus non-specific staining, which could identify false positives. However, even with the IFA test, false positive results have been observed due to cross-reactive antibodies from encephalitic patients infected with flaviviruses [29], which is a limitation to consider when performing antibody binding assays. Similarly, the false positive rate, particularly in the serum IgG ELISA, can lower the positive predictive value. Therefore, exposure history, clinical symptoms, and results from additional confirmatory testing (if available) should be taken into consideration in conjunction with the N protein ELISA. One of the limitations of our study is the availability of samples from human rabies positive cases in the U.S. [60]. From a total of 41 cases were reported in the U.S. and Puerto Rico from 2003 to 2018 [49, 60], only 16 had both CSF and serum samples available with sufficient volume to test for N protein-specific antibodies. Collaboration with laboratories in rabies endemic countries would help to improve optimization, accuracy, positive predictability and repeatability of the assay.

In addition to individual samples, we also evaluated the overall ability of the ELISA to detect N protein-specific antibodies in any sample from 16 previously diagnosed rabies cases compared to the IFA. Although the results for both IFA and ELISA are comparable, there were some instances in which one test detected an antibody response and the other did not. For example, in cases 3, 15, and 16, although IFA did not detect either IgM or IgG in CSF, ELISA was positive for IgM or IgG (Fig 5A). While cases 7 and 14 did not seroconvert for IgM nor IgG in CSF or serum by IFA, the N protein ELISA was able to detect IgG in the serum (case 7) and IgM in the CSF (case 14) of those respective patients. On the contrary, there were instances in which IFA was positive and ELISA was negative, particularly with serum IgM results (cases 4, 13 and 15) and CSF IgG results (case 4, 8). Overall, the N protein ELISA was able to detect antibodies in 15 out of the 16 rabies cases (94%), whereas the IFA detected antibodies in 13 out of 16 rabies cases. The results suggest that N protein could be sufficient for the detection of RABV binding antibodies, even though other RABV protein-specific antibodies, such as G protein-specific antibodies, could also be present.

An ELISA based detection of anti-N antibodies offers several advantages compared to the IFA test. Since the recombinant N protein used in the ELISA is purified from *E*. *coli* and antibodies are detected by a colorimetric method, it could be adopted in a hospital or resource-limited setting. In contrast, IFA requires RABV-infected cells that are not fully inactivated by standard fixation methods [37]. In addition, the ELISA does not require a fluorescence microscope, fluorescence detection reagents, or a highly trained individual to differentiate RABV-specific and non-specific fluorescence staining. In that respect, the ELISA gives an opportunity to standardize an assay globally across different labs, allowing for direct comparability of anti-N IgM and IgG kinetics. However, further validation is required and implementation of the N protein ELISA is recommended in conjunction with other standard antemortem diagnostic tests. The ELISA also offers the opportunity to test samples in a high–throughput format compared to the IFA test. Therefore, it can be optimized for non-diagnostic purposes, such as animal surveillance studies or serosurveys.

## Supporting information

S1 Fig N protein coating concentration and serum dilution optimization for IgG serum ELISA. OD values were evaluated at different serum concentrations using SRIG, serum from a positive rabies case, and serum from a negative non-rabies case using (A) 0.125 μg/ml, (B) 0.5 μg/ml, and (C) 1 μg/ml coating concentrations of recombinant N protein. SRIG served as a positive control in this test. The OD value ratio of positive to negative serum (D) and SRIG to negative serum (E) for each N concentration at each sample dilution was calculated. OD values were calculated by subtracting the blank values from observed samples values at each condition.
(TIF)

S2 Fig N coating concentration and anti-IgM secondary dilution optimization for IgM serum ELISA. OD values were evaluated at different secondary IgM antibody dilutions using SRIG, serum from a positive rabies case, and serum from a negative non-rabies case at (A) 0.125 μg/ml, (B) 0.5 μg/ml, and (C) 1 μg/ml coating concentrations of recombinant N protein. SRIG served as a negative control in this test. (D) The ratio between positive and negative serum was evaluated at each N coating concentration and secondary antibody dilution. OD values were calculated by subtracting the blank values from observed samples values at each condition.
(TIF)

S3 Fig CSF sample dilution optimization. Sample dilution for (A) IgG ELISA and (B) IgM ELISA using CSF from rabies and non-rabies diagnosed cases was determined.
(TIF)

S4 Fig Flowchart representing patient samples used for analysis. (A) Number of CSF samples and (B) serum samples from rabies cases (red) and non-rabies cases (grey) are indicated. The arrows show the number of samples used for case-by-case comparison or IFA comparison analysis.
(TIF)

S1 Table Summary table indicating sample number and clinical highlights for each rabies case. Clinical information on some cases is not available and is indicated by N/A.
(DOCX)
