# Supplementary figures and images for: An ELISA-based method for detection of rabies virus nucleoprotein-specific antibodies in human antemortem samples
Source: PLoS One. 2018 Nov 7;13(11):e0207009. doi: 10.1371/journal.pone.0207009 (PMC6221316; doi:10.1371/journal.pone.0207009)

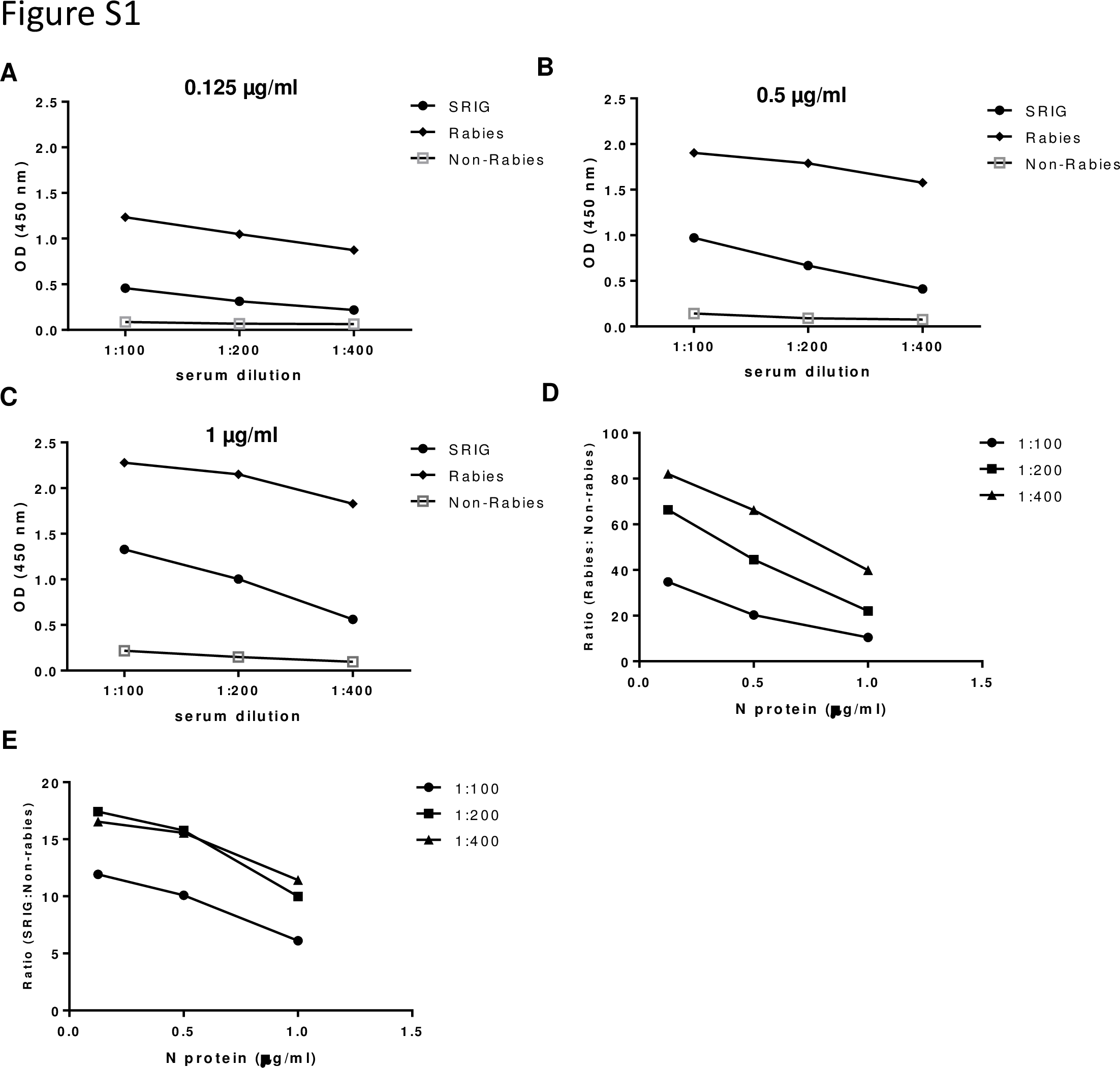

Supplement: S1 Fig — OD values were evaluated at different serum concentrations using SRIG, serum from a positive rabies case, and serum from a negative non-rabies case using (A) 0.125 μg/ml, (B) 0.5 μg/ml, and (C) 1 μg/ml coating concentrations of recombinant N protein. SRIG served as a positive control in this test. The OD value ratio of positive to negative serum (D) and SRIG to negative serum (E) for each N concentration at each sample dilution was calculated. OD values were calculated by subtracting the blank values from observed samples values at each condition. (TIF) [file pone.0207009.s001.tif]

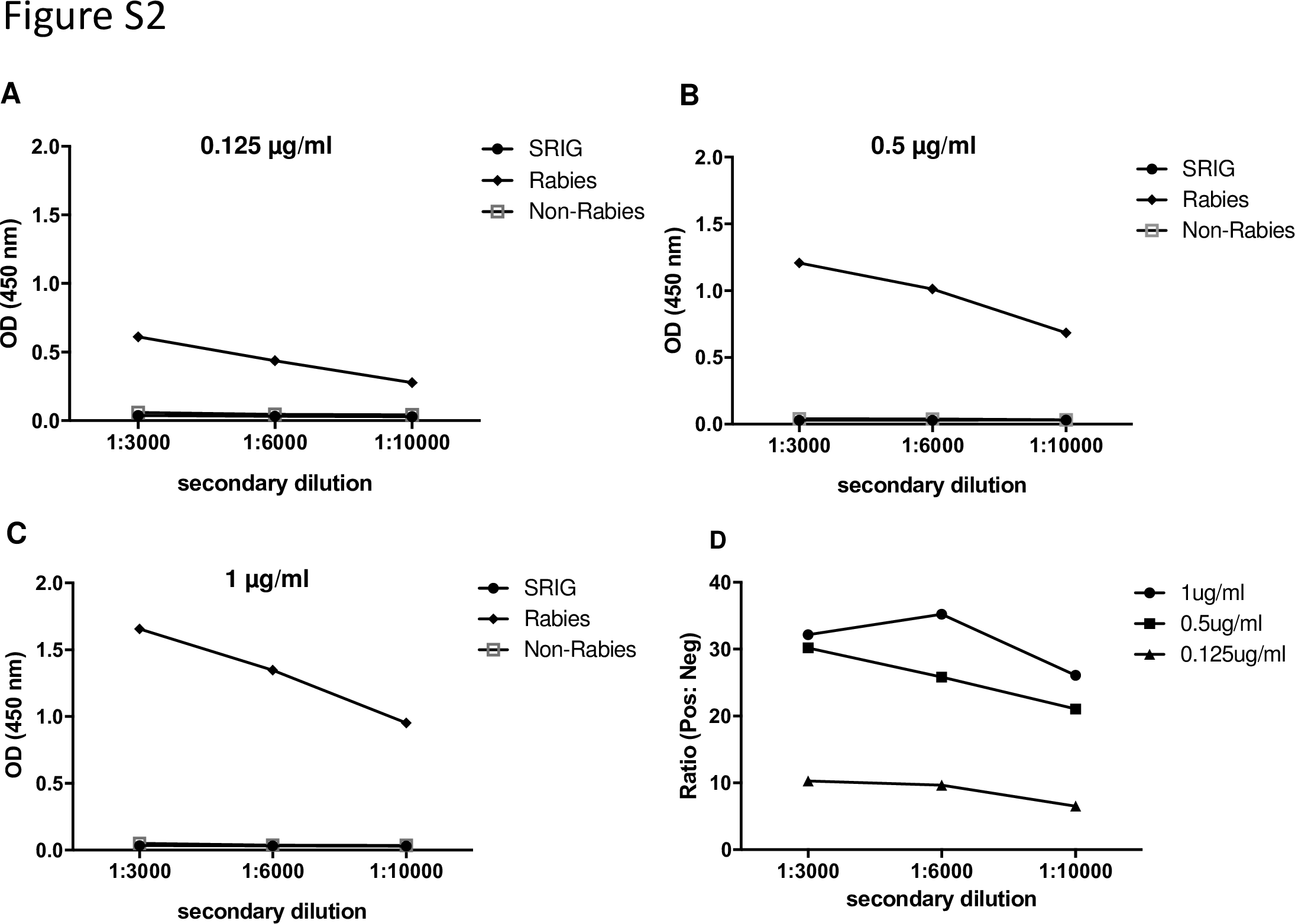

Supplement: S2 Fig — OD values were evaluated at different secondary IgM antibody dilutions using SRIG, serum from a positive rabies case, and serum from a negative non-rabies case at (A) 0.125 μg/ml, (B) 0.5 μg/ml, and (C) 1 μg/ml coating concentrations of recombinant N protein. SRIG served as a negative control in this test. (D) The ratio between positive and negative serum was evaluated at each N coating concentration and secondary antibody dilution. OD values were calculated by subtracting the blank values from observed samples values at each condition. (TIF) [file pone.0207009.s002.tif]

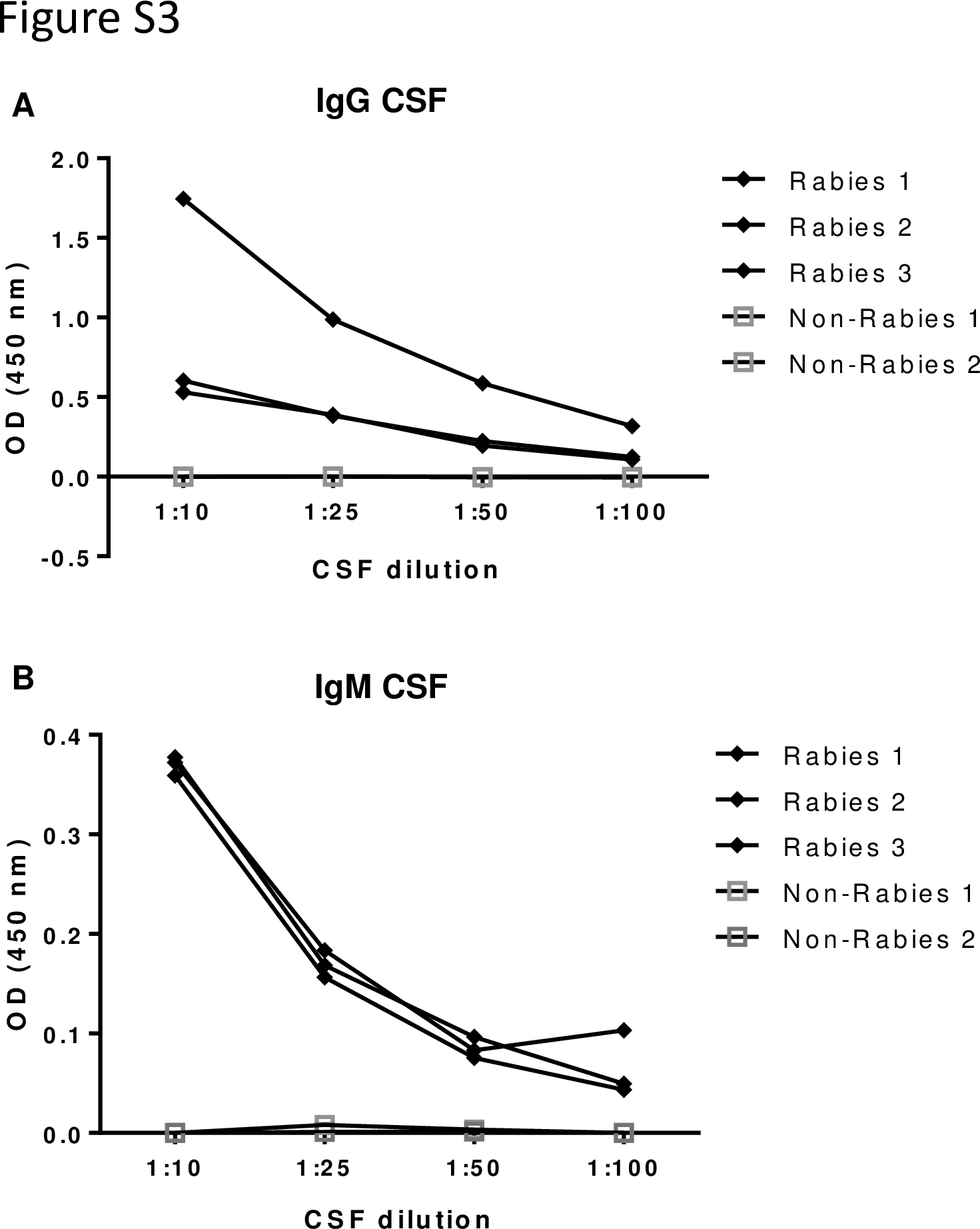

Supplement: S3 Fig — Sample dilution for (A) IgG ELISA and (B) IgM ELISA using CSF from rabies and non-rabies diagnosed cases was determined. (TIF) [file pone.0207009.s003.tif]

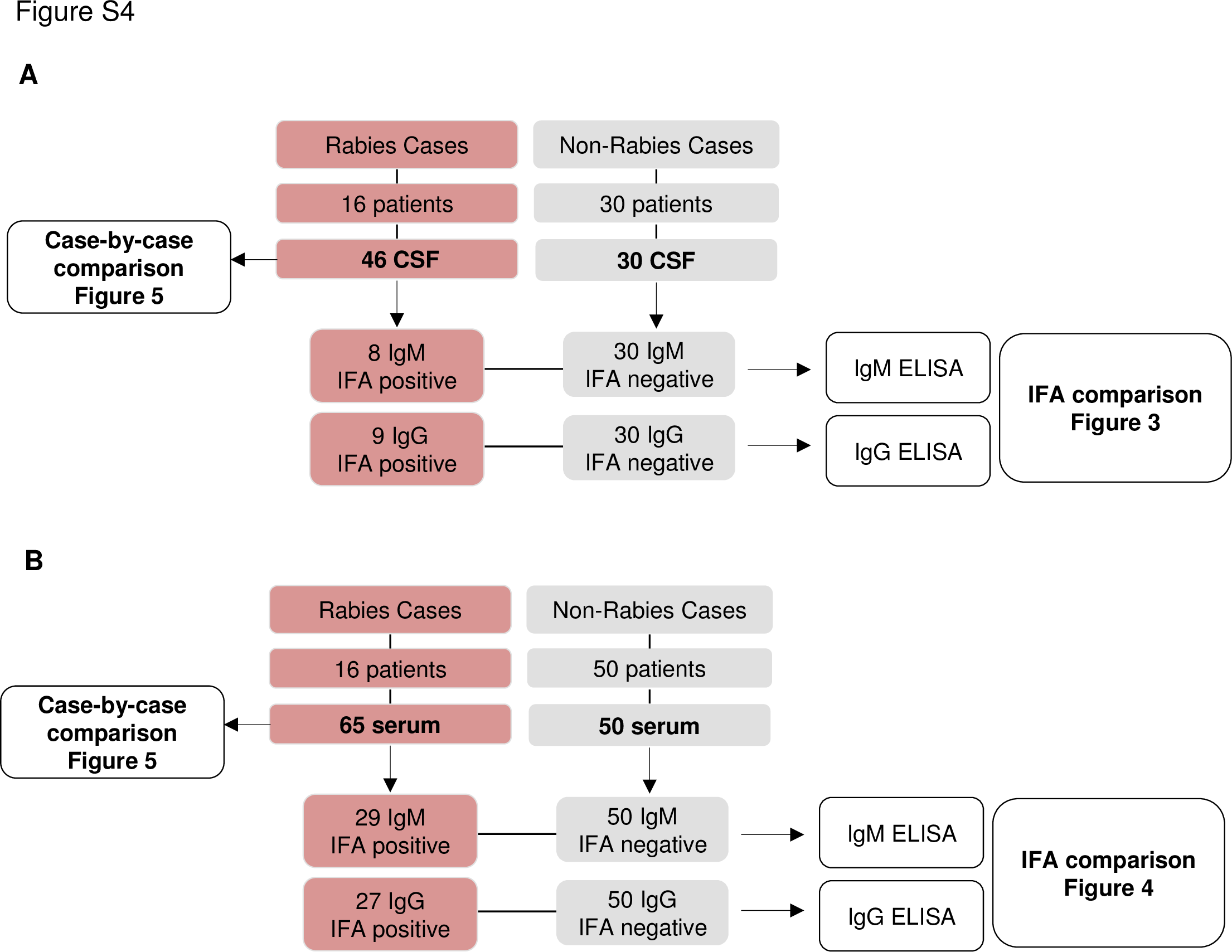

Supplement: S4 Fig — (A) Number of CSF samples and (B) serum samples from rabies cases (red) and non-rabies cases (grey) are indicated. The arrows show the number of samples used for case-by-case comparison or IFA comparison analysis. (TIF) [file pone.0207009.s004.tif]
