# Supplementary material for: An ELISA-based method for detection of rabies virus nucleoprotein-specific antibodies in human antemortem samples
Source: PLoS One. 2018 Nov 7;13(11):e0207009. doi: 10.1371/journal.pone.0207009 (PMC6221316; doi:10.1371/journal.pone.0207009)
Supplement: S1 Table — Clinical information on some cases is not available and is indicated by N/A. (DOCX) [file pone.0207009.s005.docx]

| **Samples tested by ELISA** | | | **Source** | **Clinical Highlights** |
| --- | --- | --- | --- | --- |
| **Rabies Case** | **CSF** | **Serum** |  |  |
| **1** | 0 | 2 | Bat | encephalitis, fever, paresthesia, agitation and combativeness |
| **2** | 4 | 5 | Bat | paresthesia, weakness, fever, respiratory failure |
| **3** | 14 | 13 | Dog | chills and hot flashes, Involuntary dystonic movements, localized pain |
| **4** | 4 | 4 | Bat | fever, headache, photophobia, paresthesia, agitation and combativeness |
| **5** | 1 | 1 | Bat | numbness, weakness, ataxia, difficulty swallowing and speaking, quadrlegia, comotose |
| **6** | 1 | 1 | Bat | encephalitis, localized pain, fatigue, numbness, fever, areflexia, respiratory distress |
| **7** | 0 | 1 | Bat | shortness of breath, chills, paresthesia, diaphoresis, comatose, multiorgan failure |
| **8** | 2 | 9 | Dog | paresthesias, fever, nausea, vomiting, hydrophobia, aerophobia, agitation and combativeness |
| **9** | 0 | 1 | Dog | shoulder and chest pain, headaches, hypertension, fever, combative |
| **10** | 7 | 9 | Bat | localized pain, decreased appetite, hydrophobia |
| **11** | 1 | 2 | Dog | fever, insomnia, anxiety, nausea, dyshagia, hypersalivation, agitation |
| **12** | 0 | 2 | Bat | fever, anxiety, fear, hallucinations, tremors and jerks |
| **13** | 1 | 1 | Dog | vomiting, espigastric pain |
| **14** | 4 | 6 | Dog | N/A |
| **15** | 3 | 4 | Bat | N/A |
| **16** | 4 | 4 | Bat | N/A |
